# The role of extracellular vesicles in acquisition of resistance to therapy in glioblastomas

**DOI:** 10.20517/cdr.2020.61

**Published:** 2021-03-19

**Authors:** Anudeep Yekula, Abigail Taylor, Alexandra Beecroft, Keiko M. Kang, Julia L. Small, Koushik Muralidharan, Zachary Rosh, Bob S. Carter, Leonora Balaj

**Affiliations:** ^1^Department of Neurosurgery, Massachusetts General Hospital, Boston, MA 02114, USA.; ^2^Brigham Young University, Provo, UT 84602, USA.

**Keywords:** Glioblastoma, resistance, extracellular vesicles, temozolomide, radiation

## Abstract

Glioblastoma (GBM) is the most aggressive primary brain tumor with a median survival of 15 months despite standard care therapy consisting of maximal surgical debulking, followed by radiation therapy with concurrent and adjuvant temozolomide treatment. The natural history of GBM is characterized by inevitable recurrence with patients dying from increasingly resistant tumor regrowth after therapy. Several mechanisms including inter- and intratumoral heterogeneity, the evolution of therapy-resistant clonal subpopulations, reacquisition of stemness in glioblastoma stem cells, multiple drug efflux mechanisms, the tumor-promoting microenvironment, metabolic adaptations, and enhanced repair of drug-induced DNA damage have been implicated in therapy failure. Extracellular vesicles (EVs) have emerged as crucial mediators in the maintenance and establishment of GBM. Multiple seminal studies have uncovered the multi-dynamic role of EVs in the acquisition of drug resistance. Mechanisms include EV-mediated cargo transfer and EVs functioning as drug efflux channels and decoys for antibody-based therapies. In this review, we discuss the various mechanisms of therapy resistance in GBM, highlighting the emerging role of EV-orchestrated drug resistance. Understanding the landscape of GBM resistance is critical in devising novel therapeutic approaches to fight this deadly disease.

## Introduction

Glioblastoma (GBM) is the most common primary brain tumor with a median survival of 15 months despite therapy^[1]^. The standard of care at initial diagnosis includes maximal surgical resection of the tumor followed by radiation therapy with concurrent and adjuvant temozolomide (TMZ) treatment^[2,3]^. The natural history of GBM is characterized by inevitable recurrence in 6-9 months after the initiation of therapy. GBMs are notorious for their ability to evade therapy and recur. Several researchers have focused their efforts on delineating the mechanisms underlying recurrence in GBM, with a special focus on therapy resistance. Multiple synergistic factors have been implicated in recurrence of GBM including inter- and intratumoral heterogeneity, the evolution of therapy-resistant clonal subpopulations, reacquisition of stemness in glioblastoma stem cells (GSCs), the impenetrable nature of the blood-brain barrier (BBB), multiple drug efflux mechanisms, the tumor-promoting microenvironment, metabolic adaptations, enhanced repair of drug-induced DNA damage, and tunneling tumor nanotubes^[4]^. Additionally, molecular factors such as TP53 mutation, MIB-1 labeling, and O-6-methylguanine-DNA methyltransferase have been correlated with GBM recurrence^[5,6]^. Management of recurrent GBM has been challenging with treatment options ranging from re-resection, re-radiation, systemic chemotherapy, immunotherapy, or a combination of these^[7]^. However, none of these strategies have proven beneficial. The choice of therapy is individualized depending on the time since diagnosis, radiological pattern, prior therapies, and, most importantly, the general and neurological function of the patient. Patients with recurrent GBM have a median survival of only 3-6 months^[8]^.

A series of seminal studies has uncovered the role of membrane-bound nanoparticles called extracellular vesicles (EVs) in the development of resistance in GBM. EVs are conduits for communication between cells, transporting molecular cargo including proteins, nucleic acids, and lipids from cell to cell^[9,10]^. The transfer of molecules facilitates tumor progression, angiogenesis, immune tolerance, modification of tumor metabolism, metastasis, invasion, and evasion of cell death^[9,11-13]^. Novel mechanisms such as EV-mediated drug efflux; EVs functioning as drug decoys; and EV-mediated transfer of functional mRNAs, miRNAs, long non-coding RNAs, spliceosomes, drug efflux pumps, and other resistance-acquiring products have been implicated in resistance to chemoradiation^[12]^. In this review, we explore the role of EV-orchestrated therapy in the global landscape of GBM therapeutic resistance. We emphasize the role of minimally invasive EV-based liquid biopsy platforms for monitoring recurrence, tumor evolution, and response to therapy^[14,15]^.

## Mechanisms of therapy resistance in GBM

Therapeutic resistance remains a major barrier to the successful management of GBM. Multiple synergistic mechanisms have been described to characterize the ability of GBM to evade therapy. Understanding the cellular and molecular mechanisms underlying GBM therapy resistance is critical for the management of this deadly cancer.

## Intertumoral, intratumoral heterogeneity, and clonal evolution

Elegant sequencing studies in the past decade have unveiled the heterogeneous genomic and transcriptomic landscape of GBM^[16-18]^. At the genomic level, GBMs are broadly classified as IDH-wildtype or IDH-mutant. Additional genetic alterations including but not limited to epidermal growth factor receptor (EGFR) overexpression, phosphate and tensin homolog (PTEN) mutations, p53 mutations, and loss of chromosome 10q have been implicated. At transcriptomic levels, GBMs are categorized as proneural (harboring TP53, PDGFRA, and IDH mutations), classical (harboring EGFR mutations), or mesenchymal (NF1 mutations). Despite this heterogeneity, most tumors were found to harbor alterations in core oncogenic pathways: the tumor protein p53 pathway, the receptor tyrosine kinase/Ras/phosphoinositide 3-kinase signaling pathway, and the retinoblastoma pathway^[19]^. Additionally, recent single-cell sequencing studies have provided comprehensive evidence of the intratumor heterogeneity of GBM. Neftel *et al*.^[20]^ demonstrated the presence of four specialized transcriptional subclones with varying rates of proliferation and transition within each tumor. Specifically, they identified neural-progenitor-like cells (NPC-like) with CDK4 amplifications, oligodendrocyte-progenitor-like cells (OPC-like) with PDGFRA amplifications, astrocyte-like cells (AC-like) with EGFR aberrations, and mesenchymal-like cells (MES-like) with Chr5q deletions and NF1 alterations.

Currently available therapies are not tailored to each subclone, which leads to the selection of resistant and/or untargeted subclones of cells. These selected resistant/untreated cells that have survived therapy due to their genetic makeup accumulate additional mutations and evolve into aggressive recurrent tumors^[21-24]^. This evolution of tumors can be compared to the Darwinian process of clonal selection^[25]^. Additionally, these subclones within recurrent tumors maintain differential drug resistance profiles^[26]^. Only 45% of mutations are shared between the primary and the recurrent tumor, with the dominant clone at recurrence being significantly different from the dominant clone at diagnosis^[21,27]^. This selection of clonal subtypes and accumulation of alterations can produce diverse clonal populations with series of parallel expansions within each tumor^[23]^. The spatiotemporal divergence in recurrent tumors also occurs at epigenomic and transcriptomic levels^[23,24,28]^. This evolutionarily divergent clonal selection is the crux of therapy failure and development of resistance. In this regard, the current practice of selecting a therapeutic option for a recurrent tumor based on the characteristics of the primary tumor may not be appropriate^[23]^. Therapeutic strategies should be tailored to the clonal subpopulations in order to radically target all/most cell types and prevent/minimize the selection of aggressive tumor subclones. This highlights the need for the development of multimodal poly-therapeutic strategies as opposed to monotherapies^[29]^.

## Glioblastoma stem cells

GSCs represent a group of self-sustained cells that have attained a mutational profile capable of tumorigenesis due to their ability to proliferate and self-renew^[30]^. Multiple studies have demonstrated the tumorigenic properties of GSCs^[30,31]^. These cells are classically identified based on the presence of cell surface markers such as CD133, CD44, and L1 cell adhesion molecules (L1CAM)^[32,33]^. Furthermore, GSCs modulate the tumor microenvironment supporting tumor growth and proliferation^[12]^. Despite therapy, these GSCs maintain stemness, survive, and proliferate to produce a recurrent tumor. Recent studies have demonstrated that the GSCs in recurrent GBM differ from the GSCs that initiated and maintained the primary tumor. The GSCs of recurrent tumors are more aggressive, display altered surface marker profiles (loss of CD133 and gain of CD15, BMI1, and SOX2), and correlate with shorter survival of patients in a recurrent setting^[34-36]^.

Recent studies have recapitulated the existence of quiescent and active cancer stem cells^[37]^. Researchers have hypothesized that chemoradiation targets rapidly dividing active cancer stem cells while the quiescent cancer stem cells survive chemoradiation, which eventually recapitulates the tumor^[37,38]^. Additionally, CD133+ GSCs have demonstrated chemoresistance and radioresistance. In GSCs, TMZ chemoresistance is mediated by increased expression of anti-apoptotic genes, MGMT-mediated DNA repair mechanisms, and drug efflux transporters^[39]^. Radioresistance is mediated through activation of DNA damage checkpoint and repair of radiation-induced DNA damage by arresting cell cycle^[40]^. GSCs acquire therapeutic resistance following repeated chemoradiation^[35,41]^. Recent studies have unveiled pathways involved in GSC maintenance, survival, self-renewal, and proliferation. These have been comprehensively elaborated in a recent review^[42]^. Targeting these GSCs and their associated pathways has been explored but limited in success^[43]^. Curtailing the GSC populations will be critical in developing therapeutic strategies to combat recurrence.

## BBB and drug efflux mechanisms

Achievement of therapeutic concentrations of chemotherapeutic drugs in the CNS has been a major challenge in the management of GBM. The BBB, which separates the lumen of cerebral blood vessels from the brain parenchyma, stringently regulates the entry of circulating toxins, macromolecules, and inflammatory cells. The tight junctions between the endothelial cells of CNS blood vessels in addition to the supportive pericytes and astrocytes maintain the integrity of the BBB^[44]^. GBM disrupts the integrity of the BBB, and this endothelium in contact with the GBM is referred to as the blood-tumor barrier (BTB). In GBM, the BTB has a heterogeneous permeability owing to local anatomic invasion, downregulation of tight junction proteins, upregulation of transporter proteins, and angiogenesis. The BTB is also characterized by aberrant pericyte distribution and the loss of astrocytic endfeet and neuronal connections^[45]^. Furthermore, the BBB is reinforced by efflux pumps including ATP-binding cassette efflux transporters, P-glycoprotein (P-gp), multidrug resistance protein (MRP), and breast cancer resistance protein, which actively transport out substrates^[46-49]^. Interestingly, these drug efflux transporters were also described to reduce intracellular drug accumulation in GBM cells^[46,50]^. The BBB/BTB in conjunction with BBB associated efflux transporters functions synergistically as tumor-promoting adaptive mechanisms to selectively permit the transport of tumor-supporting substances while limiting the entry of antitumor substances^[4]^. Although “leaky”, the disrupted BBB in GBM paradoxically limits the achievement of therapeutic concentrations of chemotherapeutic drugs. Additionally, altered cell adhesion molecular profiles of BBB endothelial cells have been shown to limit the transmigration of adoptively transferred T cells and chimeric antigen receptor (CAR) T cells, posing a substantial challenge^[45]^.

Several strategies have been attempted to target the BBB to enhance the delivery of chemotherapeutic agents: molecular approaches such as the use of endogenous influx transporter-mediated drug delivery; invasive approaches such as convection-enhanced delivery by direct injection, intrathecal, intraventricular injections, and implantation of wafers, gels, and microchips to bypass the BBB; and physical approaches such as magnetic resonance-guided focused ultrasound-mediated disruption of the BBB. These modalities have their drawbacks and offer limited benefit^[45]^. Co-administration of chemotherapeutic drugs in conjunction with inhibitors of BBB-associated efflux transporters have shown promise^[51-53]^. Unfortunately, all these modalities have technical limitations, inadequate CNS penetrance, systemic toxicities, and limited efficacy^[4,54]^.

## Glioblastoma tumor microenvironment

The GBM tumor microenvironment consists of glial cells (astrocytes, oligodendrocytes, ependymal cells, and microglia) and invading immune cells (macrophages, monocytes, and lymphocytes). GBM hijacks the tumor microenvironment and modifies it into a tumor-promoting environment^[55]^. These adaptations allow the tumors to survive various chemotherapeutic insults and proliferate. Researchers have attempted to harness the potential of immune cells of the TME to generate anti-tumor responses. Immunotherapy for GBM includes checkpoint inhibitors, CAR-T therapy, vaccines, and viral therapy^[55,56]^. In addition to challenges such as intra-tumoral and intratumoral heterogeneity, the tumor microenvironment provides unique hurdles to the success of immunotherapy. Immunosurveillance of the CNS is inherently adapted to maintain neuronal function and minimize non-specific immune responses. Additionally, GBM generates an immunosuppressive microenvironment by secreting soluble factors, interleukins, prostaglandins, and EVs to induce a tumor supportive M2 phenotype in tumor-associated myeloid cells (microglia, monocytes, and macrophages), induce T cell dysfunction, and suppress NK cell activity^[12,56]^. The limitations of the immune response in the CNS and the highly immunosuppressive nature of GBM limit the effectiveness of the antitumor response induced by immunotherapeutic agents.

## Metabolic adaptations

Metabolic adaptations of GBM are intrinsic to the growth and proliferation. Detailed metabolic alterations in GBM and specific pathways implicated in therapy resistance are outlined in recent reviews^[57,58]^. Targeting metabolic pathways governing GBMs adaptations has been of interest over the past decade^[58]^. However, there is growing evidence supporting the evolution of resistance mechanisms^[59]^. Rapidly growing tumors such as GBM are characterized by intratumoral hypoxia and necrosis due to deficiencies in O_2_ delivery and availability. GBM cells adapt to the hypoxic microenvironments through the activation of hypoxia-inducible factors promoting angiogenesis, induction of stem-like phenotype, chemoresistance, and motility^[60]^. Hypoxia facilitates anaerobic glycolysis and the pentose phosphate pathway, which provide survival advantage^[61,62]^. Hypoxic environments have a reduced concentration of therapeutic agents due to limited vascular supply and upregulation of drug efflux^[63,64]^. Emerging evidence suggests the presence of metabolically quiescent cells which could potentially be additional mediators of therapy resistance^[65,66]^. Interestingly, studies have highlighted the role of unfolded protein response-based stress effects in tumor-protective effects and TMZ chemoresistance in gliomas^[67]^. Thus, understanding the multidimensional role of hypoxia, angiogenesis, and metabolic alterations in resistance to therapy will provide attractive therapeutic options.

## Drug damage repair

Tumor cells depend excessively on DNA repair mechanisms to help them cope with exacerbated DNA damage resulting from increased metabolism, replication, and mitotic stress. Alkylating agents (TMZ) and ionizing radiation primarily induce DNA damage. Double-stranded breaks (DSB) inflicted by radiation are repaired by homologous recombination and non-homologous end-joining, while single-stranded breaks (SSB) are repaired by base excision repair (BER) and single-stranded repair mechanisms^[68,69]^. TMZ induces the formation of N3-methyladenine (N3-meA) and N7-methylguanine (N7-meG), cytotoxic lesions which are primarily repaired by BER, and O6-methylguanine (O6-meG), a cytotoxic lesion which is managed by O6-methylguanine-DNA methyltransferase (MGMT)^[70]^. Epigenetic silencing of MGMT promoter by methylation reduces tumoral DNA repair capacity and is associated with increased TMZ sensitivity^[71]^. In the absence of MGMT, O6-meG mispairs with cytosine or thymine. Mismatch repair machinery (MMR) recognizes the mispairs and leaves the O6-meG intact, leading to SSBs and DSBs^[21,72]^. Efficient DNA repair mechanisms and the loss of MMR activity have been implicated in GBM resistance and recurrence. Several therapeutic strategies aimed at the DNA repair axis via MGMT transcriptional regulation, MGMT silencing, and inhibition of DNA repair mechanisms are under exploration and provide attractive therapeutic targets^[73,74]^.

## Tumor microtubules

A recent study by Osswald and colleagues showed that GBM tumor cells form a functional network by extending ultralong membrane protrusions called tumor microtubules (TMs) to connect and communicate with neighboring tumor cells. TMs are implicated in tumor cell invasion, proliferation, interconnection, and radioresistance. Cytotoxic effects of radiation are largely mediated by an increase in intracellular calcium. TM-connected tumor cells have displayed resistance to the cytotoxic effects of radiation by maintaining calcium homeostasis by network integration. Additionally, TMs provide a means of effective distribution of small molecules among other cells of the network^[4,75]^. Pharmacological targeting of TM formation should be explored for the management of treatment-resistant brain tumors.

## Role of extracellular vesicles in therapy resistance

**Figure 1:**
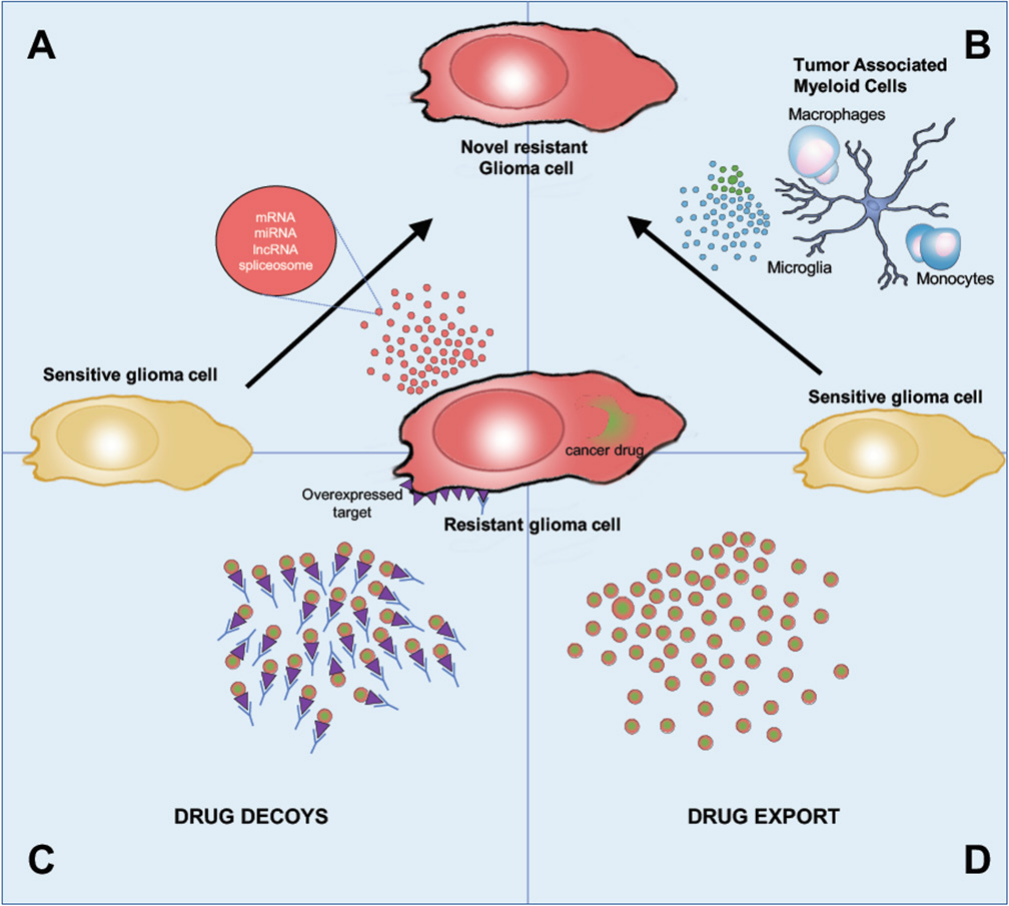
Overview of EV-mediated mechanisms of drug resistance. EVs derived from (A) resistant tumor cells and (B) tumor supporting cells transfer genomic and proteomic cargo to glioma treatment sensitive cells, which enhances their acquisition of a resistant phenotype; (C) EVs also function as decoys for antibody-based therapies, leading to the sequestration of anticancer antibodies; (D) EVs package and export drugs out of the cells, reducing its intracellular concentration

Extracellular vesicles have attracted widespread interest over the past decade for their role in modulating tumor microenvironment to promote tumor growth, proliferation, angiogenesis, therapy resistance, invasion, and evasion of immune surveillance. We elaborated on the multidimensional role of EVs in the tumor microenvironment in our recent review^[12]^. EV-mediated therapy resistance is yet another adaptation of GBM to overcome therapy. Multiple EV-mediated mechanisms of therapy resistance have been described across the literature in the context of numerous systemic cancers including breast, prostate, lung, renal, ovarian, hematologic, pancreatic, gastric, and brain cancers^[76]^. The major EV-mediated mechanisms of acquisition of therapy resistance are depicted in Figure 1.

## GBM-mediated EV transfer

Multiple studies across several systemic cancers have shown that EV-mediated transfer of functional cargo (mRNAs, miRNAs, long non-coding RNA (lncRNA), and proteins) induces drug resistance in drug-sensitive cancer cells^[76]^. The transferred functional molecules upsurge drug efflux^[77-80]^, enhance drug metabolism/inactivation^[81]^, activate anti-apoptotic and tumor-promoting pathways^[82-88]^, elicit downstream changes in signal transduction and gene expression^[89-95]^, and promote epithelial-to-mesenchymal transition^[96-99]^ to favor a resistant phenotype.

GBM cells actively modulate the composition of EVs in response to chemotherapy and radiation^[100,101]^. The resistant GBM cells produce specialized EVs with cargo capable of inducing a resistant phenotype in the recipient sensitive cells. EV-mediated transfer of multiple genomic and proteomic cargo including mRNA, miRNA, lncRNA, spliceosomes, and proteins have been reported in the context of TMZ and radioresistance in GBM^[102-108]^. The EV cargo causes a range of downstream effects in the recipient sensitive cells. Transfer of transcripts of DNA repair enzymes such as alkyl purine-DNA-N-glycosylase (APNG) and O(6)-methylguanine DNA methyltransferase (MGMT) causes increased DNA repair capability in the recipient cells^[109,110]^. Uptake of the lncRNA lncSBF2-AS was shown to enhance DNA damage repair by upregulating the lncSBF2-AS1-miR-151a-3p-XRCC4 DNA repair axis^[111]^. EV-mediated transfer of miRNAs has been demonstrated to activate anti-apoptotic pathways, enhancing GBM cell proliferation in sensitive GBM cells in response to TMZ and radiotherapy^[112-114]^.

## EVs derived from cells of the tumor microenvironment

In addition to the transfer of cargo and drug resistance mechanisms spread from resistant cells to sensitive cells, stromal cells and immune cells were also implicated in EV-mediated resistance transfer. Specifically, EVs derived from cancer-associated fibroblasts^[115-118]^, cancer-associated adipocytes^[118]^, and tumor-associated macrophages^[119,120]^ in the tumor microenvironment have been shown to impart drug resistance in sensitive cells via the transfer of functional cargo. EVs derived from GBM associated macrophages and GBM associated astrocytes were also implicated in the development of TMZ resistance and radioresistance^[109,121]^.

## EVs as drug efflux channels

Cancer cells have been shown to package and export chemotherapeutic agents in EVs. Recent studies in ovarian and pancreatic cancer-derived EVs demonstrate the presence of drug efflux transporters such as MRP and P-gp. This localization of drug efflux pumps enhances the uptake of drugs into the vesicles for eventual release^[122,123]^. The presence of transporters in reverse orientation on the surface of EVs, leading to drug import into EVs, further validated this mechanism^[76,124]^. Studies in breast cancer have shown that cell lines resistant to mitoxantrone confine the drug in EV-like structures within the cell near the cell-cell attachment regions. These EV-like structures contain drug efflux transporters that mediate intravesicular drug accumulation. However, the authors did not investigate the release of these EV-like structures^[125]^. This export of chemotherapeutic agents via EVs leads to a reduction in the intracellular concentration of the drug, reducing its efficacy and promoting resistance.

## EVs for decoys as antibody-based therapies

EVs released by cancer cells contain surface markers, which are often targets for antibody-based immunotherapies. Anticancer antibodies that target specific surface markers of interest have been demonstrated to bind to EVs containing the same surface markers. This leads to the neutralization of anticancer antibodies (anti-HER2 monoclonal antibody, trastuzumab^[125]^, and anti-CD20 monoclonal antibody, rituximab^[79]^) by competitive inhibition, thus reducing their bioavailability. This was supported by the detection of large quantities of monoclonal anti-cancer antibody-EV complexes^[79,126]^. Neutralization of anti-cancer antibodies by EVs has been shown to reduce the bioavailability of the drug by almost 50%^[79]^. Elegant studies by Simon and colleagues identified bevacizumab (monoclonal anti-VEGF antibody) on the surface of EVs derived from glioma cells treated with bevacizumab but not on EVs derived from control cells. The formation of EV-bevacizumab complexes reduces the bioavailability and the efficacy of the anti-angiogenic drug *in vitro*. Furthermore, inhibition of EV production increased the efficacy of the drug, validating the role of EVs as drug decoys. However, these mechanisms have not yet been validated *in vivo* and the downstream effects of EV-bevacizumab complexes are still unknown^[127]^.

Detailed descriptions of studies describing EV-mediated mechanisms of EV resistance in GBM are outlined in Table 1. In summary, the transfer of these factors enhances therapy resistance by a reduction in intracellular concentration, inactivation of chemotherapeutic drugs, activation of antiapoptotic pathways, upregulation of tumor-promoting pathways, and promoting a switch to a mesenchymal phenotype. These phenomena can be considered additional ways of intercellular communication between resistant and sensitive cancer cells to overcome therapy. They provide insight into the tremendous capability of the GBM cells to dynamically modulate EV composition based on the noxious stimulus they are exposed to. This principle can be broadly applied to the multitude of stresses that cancer cells face, as well as their effective communication and coping mechanisms to overcome these hurdles. Interestingly, multiple studies showed that treating resistant cells with EVs carrying drug sensitizing cargo induced chemosensitivity in the recipient cells^[128,129]^, indicating the impact of transferred functional cargo and the potential role of EVs as drug delivery tools. Understanding the role of EVs in the acquisition of resistance is important to target EV-mediated escape mechanisms.

**Table 1:** Summary of studies describing EV-mediated mechanisms of EV resistance in GBM

Author, year	Therapy	Mechanism of EV mediated resistance transfer	Genetic cargo evaluated	Functional implication	Validation: *in vitro*/*in vivo*
Zhang *et al.*^[111]^, 2019	TMZ	EV mediated cargo transfer	lncRNA, lncSBF2-AS	Enhanced DNA damage repair by upregulating lncSBF2-AS1-miR-151a-3p-XRCC4 DNA repair axis	Both
Yin *et al.*^[112]^, 2019	TMZ	EV mediated cargo transfer	miRNA, miR-1238	Anti-apoptotic function by the activation of EGFR-PI3K-Akt-mTOR pathway	Both
Chuang *et al.*^[121]^, 2019	TMZ	GBM associated macrophage EV mediated cargo transfer	miRNA, miR-21-5p	Enhanced survival by modulating tumor suppressor gene, PDCD4 and enhancing STAT3/JAK 2 pathway	Both
Zeng *et al.*^[104]^, 2017	TMZ	EV mediated cargo transfer from (PTPRZ1-MET-ZM fusion positive cells)	Specific cargo not identified	-	Both
Munoz *et al.*^[114]^, 2019	TMZ	EV mediated cargo transfer	miRNAs, miR-93, miR-193	Decrease cell cycling quiescence by targeting Cyclin D1	*In vitro*
Yu *et al.*^[109]^, 2018	TMZ	GBM associated Astrocyte EV mediated cargo transfer	mRNA, MGMT	Transfer of MGMT mRNA increases DNA repair enzymes in recipient cells	Both
Shao *et al.*^[110]^, 2015	TMZ	EV mediated cargo transfer	mRNA, MGMT and APNG	Transfer of MGMT, APNG mRNA increases DNA repair enzymes in recipient cells	*In vitro*
Pavlyukov *et al.*^[105]^, 2018	Radiation, TMZ, Cisplatin	EV (Apoptotic) mediated cargo transfer	Spliceosome, RBM11	RBM11 switches splicing of MDM4 and Cyclin D1	Both
André-Grégoire *et al.*^[108]^, 2018	TMZ	EV mediated cargo transfer	Proteolytic and mRNA processing proteins, adhesion related proteins	-	*In vitro*
Mrowczynski *et al.*^[113]^, 2018	Radiation	EV mediated cargo transfer	Upregulated: miRNA, miR-889 mRNA, WWC1 Downregulated: miRNA, miR-365	Upregulated miR-889 (inhibits DAB2IP expression), mRNA WWC1, and downregulated miR-365 (disinhibiting expression of Cyclin-D1, BCL-2, and PI3K and PTEN) increases radioresistance	Both
Zhang *et al.*^[102]^, 2020	Radiation	GBM associated macrophage EV mediated cargo transfer	miRNAs, miR-27a-3p, miR-22-3p and miR-221-3p	Promoted proneural to mesenchymal transition by targeting CHD7 pathway	Both
Yue *et al.*^[103]^, 2019	Radiation	Hypoxia induced EV mediated cargo transfer	miRNA, miR-301a	Activates Wnt/β-catenin Signalling and inhibiting TCEAL7	*In vitro*
Dai *et al.*^[107]^, 2019	Radiation	EV mediated cargo transfer from AHIF positive cells	Specific cargo not identified	AHIF-mediated p53 downregulation and anti-apoptosis	*In vitro*
Ramakrishnan *et al.*^[106]^, 2020	Radiation	miRNA export	miRNA, miR-603	miR-603 export causes de-repression of IGF1, IGF1R and MGMT leading to radioresistance and TMZ resistance	Both
Simon *et al.*^[127]^, 2018	Bevacizumab	Decoys	-	Reduced bioavailability of bevacizumab	*In vitro*

AHIF: antisense transcript of hypoxia-inducible factor-1α; APNG: alkyl purine-DNA-N-glycosylase; EV: extracellular vesicles; GBM: glioblastoma; IGF1: insulin-like growth factor 1; IGF1R: insulin-like growth factor 1 receptor; MGMT: O(6)-methylguanine-DNA methyltransferase; TMZ: temozolomide

## Role of extracellular vesicles in tracking the evolution of resistance

In the dynamic setting of the GBM disease course, imaging modalities are limited in providing molecular information about the intratumoral heterogeneity and clonal evolution in the setting of therapy resistance. Liquid biopsy-based modalities have been explored as a potential way to provide information regarding the genomic, transcriptomic, and proteomic changes that occur in a tumor over the course of time and therapy^[130-132]^. Emerging evidence from several liquid biopsy studies showed higher quantities of resistance mediators can be detected in serum EVs of cancer patients who did not respond to chemotherapy compared to responders^[77,81,133-135]^. The dynamic role of liquid biopsy-based strategies in monitoring the course of GBM has been elaborated in recent reviews. The biomarker potential of EV-based mediators of GBM resistance described in Table 1 needs to be evaluated. This could provide an opportunity to longitudinally track the evolution of the tumor over disease course to strategize therapies based on the tumor’s behavior in response to therapy. Furthermore, liquid biopsy may also have the potential to guide clinical trials to recruit patients with recurrent GBM based on the molecular state of the recurrent tumors rather than the treatment-naive primary tumor.

## Current status of therapy

In GBM, the development of resistance to treatment and thus disease recurrence and progression is a foregone conclusion. However, there are no standardized treatment paradigms for recurrent or progressive GBMs. Current treatment strategies for recurrent GBM use multimodal strategies that focus on the debulking of symptomatic tumors, the cytotoxic effects of radiation and chemotherapy, and targeted treatment with RTKs and immunotherapies^[136-138]^. However, each of these strategies has pitfalls that have averted durable disease control.

Reoperation for GBM can be helpful to confirm disease recurrence, sample the current molecular profile of the tumor, and relieve symptomatic mass effect. Resection allows for a reduction in clonal diversity within the tumor; however, less than 25% of patients undergo a second operation due to tumor location or poor prognostic factors such as low Karnofsky performance status^[139]^. In the event of reoperation, the extent of resection is a predictor for overall survival; however, a multicenter study of 503 patients undergoing re-resection for recurrent GBM demonstrated that complete and near-complete (≥ 90%, < 100%) extent of resection was reduced in reoperation as compared to initial operation^[140]^. There is evidence to suggest local strategies, such as carmustine wafers placed in the resection cavity, improve survival in patients with local recurrence; however, the adverse effects associated with wafer removal and their long-term benefits are still being evaluated^[141]^. Local control of the tumor in the surgical site has also been attempted using 5-aminolevulinic acid (5-ALA)-based photodynamic therapy, but with variable efficacy^[142,143]^. Radiation therapies, including stereotactic radiosurgery (i.e., high-dose radiation delivered in one dose), radiotherapy (i.e., fractions of radiation delivered over multiple doses), and brachytherapy (i.e., direct delivery of radiation treatment via implantable devices), have also been explored for the treatment of recurrent GBM^[144-147]^. However, these therapies are limited by maximal dose, radiation-induced toxicity, and risk of complications^[136]^. Additionally, GBMs can develop resistance to radiation therapies due to activation and adaptation of DNA repair pathways^[42,148]^.

Systemic therapies, including chemotherapeutics, targeted agents, and immunotherapies have also had limited efficacy in recurrent GBM. Bevacizumab, an anti-angiogenic monoclonal antibody targeting vascular endothelial growth factor A (VEGFA), now often used as a first-line agent for recurrent GBMs, first showed promise for the treatment of recurrent GBM in 2009 and 2010, when a series of studies assessed its use in mono- and combination therapy regimens. Although recurrent GBMs demonstrate a radiographic response to bevacizumab, the drug is not associated with increased overall survival. However, many practicing clinicians regard its positive effects such as potential sparing of steroid dose and some neurologic improvement^[149-152]^. Resistance to bevacizumab and other anti-angiogenic agents may be due to the complex network and crosstalk between different RTKs^[138]^ Furthermore, GBMs are thought to escape angiogenic growth mechanisms via upregulation of hypoxic growth factors, tumor invasion, and necrosis^[153,154]^. Nitrosoureas, such as carmustine and lomustine, are often used as second-line agents for recurrent GBM; however, the development of resistance and toxicity profiles limit the applicability of these drugs^[153,155]^. Patients with recurrent GBM can also be re-challenged with TMZ. Similar to nitrosoureas, this strategy often fails due to hypermutation or activation of DNA repair pathways which circumnavigate TMZ’s mechanism of action^[153]^. Immunotherapy modalities including checkpoint inhibitor therapies, CAR-T cell therapy, and vaccine trials have been increasingly explored. The brain immunology and accompanying therapeutic targets are masterfully discussed in a recent review by Sampson *et al*.^[137]^. The efficacy of these immunotherapeutics has been limited, which is attributable to the overall poor immunogenicity of the CNS, heterogeneous expression of immune-targetable antigens, and tumor evolution over time^[137,138]^. Oncolytic viruses are also employed to selectively infect tumor cells - inducing virus-mediated cell death as well as promoting secondary immune response - and have shown promise^[156]^. For each of these strategies, clones with innate resistance or acquired resistance promote disease recurrence and progression^[42]^.

## Future directions

Overall, future treatments will need to address intratumoral heterogeneity, treatment escape mechanisms, and microenvironmental influences to circumnavigate disease progression and allow for the creation of standardized protocols for the treatment of recurrent and progressive GBM. Understanding the myriad reasons that lead to treatment failure in GBM, including the inability to obtain a complete resection, challenges of drug delivery and crossing the BBB, limitations in clinical trial design and execution, intertumoral and intratumoral heterogeneity, reacquisition of stemness in GSCs, dynamics of the tumor microenvironment, metabolic adaptations, and the role of EVs in therapy resistance, are critical in developing targeted therapies. Lack of proper *in vivo* models, limited knowledge on drivers of progressive disease, resistance mechanisms, and treatment-induced molecular and genetic diversity have hindered the growth at the GBM therapeutic front. Liquid biopsy-based strategies could further help us understand tumor heterogeneity and the evolution of the genomic architecture of the tumor. This knowledge is essential for integrating precision diagnostics into personalized therapeutics. As such, multimodal treatment can be dynamic and tailored to the evolutionary landscape of the tumor, thus minimizing the development of resistance and ensuring a durable treatment response.

## Conclusion

Personalized therapeutic strategies complementing the evolving molecular landscape of the tumor are essential to overcome recurrence and resistance. Multidimensional approaches such as combinations of chemotherapy, radiation, and immunotherapy are critical to curtailing the cycle of therapy and resistance. Understanding the dynamic role of EVs in enhancing resistance to therapy can provide novel therapeutic targets. Targeting EV-mediated mechanisms of resistance might supplement the existing therapeutic modalities. Additionally, liquid biopsy-based monitoring can supplement therapeutic efforts by providing real-time insights into the emerging intratumoral heterogeneity of the tumor, over time and therapy.

## Declarations

### Copyright

© The Author(s) 2021.
